# The range of protein hydrolysis and biogenic amines content in selected acid- and rennet-curd cheeses

**DOI:** 10.1007/s11696-018-0490-y

**Published:** 2018-05-02

**Authors:** G. Bonczar, M. Filipczak-Fiutak, A. Pluta-Kubica, I. Duda, M. Walczycka, L. Staruch

**Affiliations:** 1 Department of Animal Products Technology, Faculty of Food Technology, University of Agriculture in Krakow, Krakow, Poland; 2 Department of Fermentation Technology and Technical Microbiology, Faculty of Food Technology, University of Agriculture in Krakow, Krakow, Poland; 3 Department of Gastronomy and Food Consumption, Faculty of Food Technology, University of Agriculture in Krakow, Balicka 122, 30-149 Krakow, Poland; 4 Department of Food Science and Technology, Faculty of Chemical and Food Technology, Slovak University of Technology in Bratislava, Radlinského 9, 812 37 Bratislava, Slovakia

**Keywords:** Acid-curd cheese, Rennet-curd cheese, Fried cheese, Biogenic amines, Toxicity

## Abstract

The aim of this experiment was to assess the range of proteolysis and biogenic amine content in the selected rennet- and acid-curd cheeses available on the Polish market, randomly chosen for the analyses: three ripened hard with eyes cheeses, three ripened hard smooth cheeses, three ripened soft moulded cheeses, three fresh unripened acid-curd cheeses, three ripened acid-curd cheeses with slimming bacteria—fried, and three ripened acid-curd cheeses with smear bacteria. The results allowed calculating the subsequent indexes and depth of proteolysis. It was found that the acid-curd short ripened cheese (harzer) was characterized by the greatest range of proteolysis and the biogenic amine content in comparison to other rennet- and acid-curd cheeses. In the assessed acid- and rennet-curd cheeses, the dominant amines were as follow, cadaverine, tyramine, and putrescine, with the exception of cheddar in which the histamine was predominant.

## Introduction

The chemical composition, physical, organoleptic, and nutritional properties of cheeses result from the influence of many factors, such as raw material (milk) quality (Delgado et al. 2011), technological processes involved in the production (Fuentes et al. 2015; Kondyli et al. 2016; Sulejmani and Hayaloglu 2016; Masotti et al. 2017), and some biochemical changes (McSweeney and Sousa 2000; Wang et al. 2011; Hong-Xin et al. 2015).

Proteins in cheeses undergo proteolysis evoked by native milk proteases (mainly plasmine), coagulum enzymes (rennine); proteases, peptidases, decarboxylases, deaminases, and other enzymes of microbiological origin (natural, from starter cultures, additional, specific—moulds, propionic bacteria, smear bacteria, and of these from the environment). The formation of medium- and low-molecular-size peptides, amino acids and their products a.o. amines, acids, aldehydes, ketones, ammonium, hydrogen sulphide, and many others from the initial casein fractions is caused by the cooperative activity of these enzymes (McSweeney and Sousa 2000; Upadhyay et al. 2004).

The proteolytic processes change the composition of cheeses, and enhance formation of bioactive peptides, free amino acids, aroma substances, and of other compounds influencing the flavour of cheeses and change the nutritional value of cheeses. This process also leads to the increase of the concentration of the substances harmful to human health, i.e., biogenic amines (Wang et al. 2011; Nega and Moatsou 2012; Hong-Xin et al. 2015).

The size of proteolysis range is described by the proportion of water soluble nitrogen in total nitrogen content in cheeses (WSN/TN) and the proportion of ammonium nitrogen in total nitrogen content (AN/TN). Different levels of these coefficients are reported, in relation to the kind of cheese, technology of production, way, and time of ageing, etc. Higher values of these coefficients are found in rennet-curd (Kondyli et al. 2016), the so-called natural, and in mould and smear-ripened cheeses than in hard cheeses ripened with bacteria (Fritsch et al. 1992; Sanchez et al. 2001; Nega and Moatsou 2012). In ripened blue-veined cheeses, WSN/TN ratio can reach about 73% (Masotti et al. 2017); in surface mould ripened cheeses (camembert) from 12 to 20% (Upadhyay et al. 2004), in semi hard and hard cheeses, i.e., cheddar (Wang et al. 2011), edam (Rabie et al. 2015), gouda (Cichosz et al. 2005), brined cheeses (Nezhad Razmjoui Akhgar et al. 2016), it ranges from 10.6 (kashkaval) (Sulejmani and Hayaloglu 2016), to 26.5% (ras cheese) (Awad 2006). Little is published on ammonium compounds in cheeses. Chen et al. 2012 reported that this content can range up to 2.14 mg g^−1^ of total solids.

Among fermented food products, cheese is most commonly related with biogenic amines (mostly histamine, tyramine, cadaverine, and putrescine) intoxication (El-Zahar 2014). The biogenic amines originating from free amino acids undergo detoxification in the human body, but, when consumed in abundance, they can be the cause of disturbances of human body functions or even food poisoning; they can also be nitrificated to cancer-causing nitrosoamines. They can evoke cardiovascular, digestive, and breathing systems diseases, show pro-cancerogenic activity, and sometimes favour very specific diseases, such as schizophrenic depression, Reyes’ syndrome, and Parkinson’s disease. (Karovičová and Kohajdová 2005, European Food Safety Authority 2011, Benkerroum 2016). There were consumption thresholds proposed for fish and other foodstuff: for histamine 50–200 mg kg^−1^ of foodstuff. Hence, in one meal, the threshold for histamine cannot exceed 50 mg, for tyramine 600 mg; however, for other bioamines, the limits are not established (European Food Safety Authority 2011).

The consumption of cheeses in Poland concerns mainly tvorogs—not ripened acidified curd (over 60% of consumption), and other cheeses like: rennet-curd cheeses with eyes; moulded (with surface and marbling moulds); hard cheeses (parmesan and cheddar); smear-ripened; and also acidified and ripened (fried, harzer).

The aim of this experiment was to assess the range of proteolysis and biogenic amine content in selected rennet- and acid-curd cheeses available on the Polish market.

## Experimental

18 cheeses for the assessment were supplied by manufacturers present at Cracow’s (Poland) market: 9 rennet-curd cheeses, 9 acid-curd cheeses. For the analyses, there were randomly chosen three ripened hard with eyes cheeses (emmental type), three ripened hard smooth cheeses (cheddar type), three ripened soft moulded cheeses (camembert type), three fresh unripened acid-curd cheeses (tvorog type), three ripened acid-curd cheeses with slimming bacteria—fried (fried cheese type), and three ripened acid-curd cheeses with smear bacteria (harzer type).

The content of total solids in cheeses was estimated with the use of the following methods: water content by drying in 130 ± 1 °C for 1.25 h using a laboratory drying oven (SML 48/250, Zalmed, Poland), fat content by Van Gulik method using a Gerber centrifuge (SuperVario N, Funke-Dr.N.Gerber Labortechnik GmbH, Germany), the total nitrogen (TN) content by Kjeldahl, protein content (TN × 6.38), the contents of water soluble nitrogen (WSN, in mineralized water filtrate), and ammonium nitrogen (AN, in directly distilled water filtrate) by Kjeldahl using Büchi Digestion and Distillation Units (K-435 and B-324, Switzerland). pH of cheeses was also obtained by conductometric measurements (CP-411, Elmetron, Poland). All the above analyses were performed according to AOAC methods (1990) and ISO 3433:^2008^. The total aerobial bacteria count was performed on samples prepared according to ISO 6887-5: 2010 and BIOCORP was used: Standard Methods LAB- AGARTM Nr PS-37 to enumerate microorganisms with ISO 13559:2002 standard.

Biogenic amines: tyramine, tryptamine, histamine, putrescine, cadaverine, spermine, spermidine, and 2-phenylethylamine amounts were obtained through RP-HPLC method according to Innocente et al. (2007), with some modifications. The solution of standards of eight biogenic amines was prepared in 0.1 M HCl in concentration of 1 mg cm^−3^ of every amine—each of the following reference substances was dissolved in 10 cm^−3^ of 0.1 M HCl:

- tyramine hydrochloride (Sigma-Aldrich, Switzerland)—12.7 mg,
- tryptamine hydrochloride (Sigma-Aldrich, USA)—12.3 mg,
- histamine dihydrochloride (Sigma-Aldrich, China)—16.6 mg,
- putrescine dihydrochloride (Sigma-Aldrich, Switzerland)—18.3 mg,
- cadaverine dihydrochloride (Sigma-Aldrich, Switzerland)—17.1 mg,
- spermine tetrahydrochloride (Sigma-Aldrich, Switzerland)—17.2 mg,
- spermidine trihydrochloride (Sigma-Aldrich, Switzerland)—17.5 mg,
- 2-phenylethylamine hydrochloride (Sigma-Aldrich, Japan)—13.0 mg.

Prepared solution of standards was then 100-fold diluted.

Derivatisation procedure: 1 cm^3^ of dansyl chloride solution in acetone (5 mg cm^−3^) and 0.5 cm^3^ of saturated NaHCO_3_ were added to 1 cm^3^ of the solution of standards. The mixture remained in darkness for 1 h in 40 °C with occasional shaking. Then, the derivatised amines were extracted twice with 1 cm^3^ of diethyl ether. The extracts were combined and dried under nitrogen flow. The precipitate was dissolved in 1 cm^3^ of acetonitrile.

Extraction of biogenic amines from cheese: 15 cm^3^ of 0.1 M HCl was added to 10 g of grated cheese, homogenised in room temperature for 2 min with 10,000 rpm (Unidrive 1000D, M. Zipperer GmbH, Germany), and centrifuged for 20 min in 4 °C with 14,000 g. The obtained supernatant was collected and filtered, and the residue was re-extracted by 15 cm^3^ of fresh 0.1 M HCl. The volume of the combined supernatants was completed to 50 cm^3^ by 0.1 M HCl. 1 cm^3^ of the extract was then derivatised by the same procedure as the solution of standards.

RP-HPLC analysis was performed with Dionex UltiMate 3000 (Thermo Scientific, USA), equipped with gradient four-component pump LPG-3400SD, autosampler ACCT-3000T, and UV–Vis detector VWD-3400RS set at 254 nm. The column was Kromasil 100-5-C18 (4.6 × 250 mm, Akzo Nobel, USA) thermostated in AT 30 °C. The elution programme was performed according to Moret et al. (2005) for DCl derivatives. The limit of detection (LOD) was 0.002 μg cm^−3^, while the limit of quantification (LOQ)—0.01 mg kg^−1^. All reagents used were HPLC grade (Sigma-Aldrich, USA).

Based on the results, the subsequent indexes of proteolysis were calculated: total nitrogen content (TN); water soluble nitrogen content (WSN); ammonium nitrogen content (AN); range of proteolysis as ratio: WSN/TN; depth of proteolysis as ratio: AN/TN (Upadhyay et al. 2004).

All cheeses samples were analysed in triplicates and each estimation was repeated three times on each single sample. Arithmetic mean values and standard deviations for all obtained data were calculated. The ANOVA (factor—cheese type) was calculated with the significance level at @@*p* ≤ 0.05. The Duncan test was used for the post hoc comparisons. The correlation coefficients were calculated for all analysed individual bioamine contents vs. basic cheeses composition, pH, bacteria count, water soluble nitrogen, and ammonium nitrogen content. The data were processed with licensed software Statistica v.11.0 (Statsoft Poland).

## Results and discussion

The analysed cheeses differed statistically (*p* ≤ 0.05) in content of water, fat, total nitrogen compounds, and pH levels (Table 1). The content of water in the analysed cheeses was comparable to those reported in the literature. According to O’Brien and O’Connor (2004), cheddar and emmental contain approximately 36% of water, while camembert approximately 51%. Dmytrów et al. (2007) and Schulz-Collins and Senge (2004) reported that tvorog can contain from 56 to 82% of water. The amount of water in analysed acid-curd ripened cheeses (fried and harzer), produced of tvorog, was smaller than in tvorog. According to Strnadová et al. (2012), Olomouc cheeses, in which technological production process is close to harzer cheeses, contain about 62% of water.

**Table 1:** Properties of cheeses ($\bar{x}$ ± sd; mean values ± standard deviation)

Property	Cheese					
	Rennet-curd			Acid-curd		
	Cheddar	Emmentaler	Camembert	Tvorog	Harzer	Fried
Water (%)	34.59^d^ ± 0.50	36.77^d^ ± 0.32	47.24^c^ ± 2.00	72.18^a^ ± 0.50	61.31^b^ ± 1.73	60.46^b^ ± 4.10
Fat (%)	33.67^a^ ± 0.88	29.00^a^ ± 0.58	30.33^a^ ± 3.18	3.27^c^ ± 1.27	0.13^c^ ± 0.07	13.73^b^ ± 2.37
Protein (%)	24.44^b^ ± 0.81	25.96^b^ ± 0.15	18.17^c^ ± 0.46	19.68^c^ ± 1.66	30.06^a^ ± 1.38	19.41^c^ ± 1.88
pH	5.53^bd^ ± 0.26	6.44^b^ ± 0.04	7.11^ab^ ± 0.26	4.66^c^ ± 0.10	7.66^a^ ± 0.04	6.06^bd^ ± 0.04
TBC (log cfu g^−1^)	7.66 ± 1.39	7.44 ± 1.34	8.30 ± 1.95	7.53 ± 1.10	8.79 ± 2.73	6.04 ± 1.94

*TBC* mesophilic aerobial count, *cfu* colony-forming unit

^a–d^Statistically significant differences between means (*p* ≤ 0.05) marked with different letters in rows

The largest amounts of fat were observed in rennet-curd cheeses (cheddar, camembert, and emmental), whereas the smallest—in harzer cheese which is produced from skimmed tvorog (Table 1). O’Brien and O’Connor (2004) described that cheddar cheeses can contain from about 26–35% of fat, whereas emmental about 29% and camembert from about 22–24%. Dmytrów et al. (2007) and Schulz-Collins and Senge (2004) stated that fresh tvorog cheeses contain from about less than 1.8 to 20% of fat. According to Esatbeyoglu et al. (2016), harzer cheese contains less than 5% of fat in dry matter.

The highest content of protein was found in harzer cheese—30.06% (Table 1). According to Esatbeyoglu et al. (2016), this kind of cheese contains 30% of protein. The amounts of these compounds in other analysed cheeses were comparable to those described by O’Brien and O’Connor (2004), according to them cheddar contains, at average, about 25.5%, emmental about 28.7%, and camembert about 20.9%, of protein, respectively. Dmytrów et al. (2007) reported, as cited in other papers, that protein content in tvorogs ranged from 12 to 20%. However, according to German regulations, in quark, it ranged from 6.8% (double cream) to above 12% (skimmed) (Schulz-Collins and Senge 2004).

The assessed cheeses showed differentiated level of pH: from 4.66 (tvorog) to 7.66 (harzer). Dmytrów et al. (2007) and Jasińska et al. (2010) found the tvorog pH ranking from 4.4 to 4.8 and it was close to isoelectric point of casein. Cheddar, due to cheddaring process resulting in, among others, the acidification of cheese curd, showed also the low pH values, but a little higher than tvorog, that is from 5.0 to 5.3 (Wang et al. 2011; Hong-Xin et al. 2015). The pH of assessed emmental (6.44) was a bit lower than that of fresh milk which resulted from the technological process applied, and long cheese curd ripening time.

The ripened camembert showed the pH value close to alkaline, which is caused by change of lactic acid into carbon dioxide, with the assistance of moulds. The similar effect is obtained when producing harzer cheese, where the changes of lactic acid are caused by smear bacteria and yeasts. The pH of fried cheese was higher than for tvorog used for its preparation, and resulted from the changes of a part of lactic acid into carbon dioxide, and then from the inhibition of the process by high temperature (during frying). According to Strnadová et al. (2012), pH of Olomouc cheeses ranges from 6.75 to 7.16.

Viable microorganisms were present in all the assessed cheeses amounting from 6.04 log cfu g^−1^ (fried cheese) to 8.79 log cfu g^−1^ (harzer) (Table 1). Owing to the great variability of the results obtained for the total aerobial microorganisms count for the examined cheeses, the differences were not statistically significant.

The amount of microorganisms in cheeses depends on many factors, among others fresh milk quality, technological processes applied (pasteurization, starter cultures addition, time and temperature of thermo-mechanical curd treatment, salting), time and temperature of ripening, and many others (Beresford and Williams 2004; Es’haghi Gorji et al. 2014; Fuentes et al. 2015; Flasarová et al. 2016). Fuentes et al. (2015) and Beresford and Williams (2004) observed that the count of microorganisms in ripened cheeses was at about 8 log cfu g^−1^.

The WSN/TN ratio is an index of protein hydrolysis (Pillonel et al. 2003; Upadhyay et al. 2004). The amount of soluble nitrogen differed with the lowest value of WSN and WSN/TN ratio in tvorog and with the highest in harzer cheese (Table 2). The value of WSN obtained for harzer cheese differed statistically (*p* ≤ 0.05) in comparison to other cheeses. The high value of WSN/TN ratio was observed for harzer, fried, and camembert cheeses. According to Nezhad Razmjoui Akhgar et al. (2016), WSN/TN ratio grows in cheeses parallel to elongation of their ripening time. Subsequent values are presented for this index in scientific papers: in blue cheeses from 17 to 73% (Masotti et al. 2017), in camembert from 9 to 20% (Upadhyay et al. 2004), in cheddar and other hard cheeses from at about 12 to about 26.5% (Pillonel et al. 2003; Awad 2006; Wang et al. 2011), in mozzarella about 5% (Nezhad Razmjoui Akhgar et al. 2016), and in acid-curd cheeses 12% (Fritsch et al. 1992). Little has been published about the WSN/TN ratio in acid-curd ripened cheeses (harzer and fried). The data obtained in our measurements (Table 2) showed that the ratio WSN/TN was higher in acidified ripened cheeses than in long time ripened rennet-curd cheeses (emmental and cheddar), so the acid-curd ripened cheeses (harzer and fried) were characterized by a greater extent of proteolysis than rennet-curd cheeses ripened for different time periods. WSN/TN ratio in fresh, unripened acid-curd tvorog cheese was comparable to the ratio calculated for long ripened rennet-curd emmental. It can be concluded that when comparing cheeses different in their production technology, the time of ripening is not the predominant factor influencing on the hydrolysis process.

**Table 2:** Proteolysis index in cheeses ($\bar{x}$ ± sd; mean values ± standard deviation)

Characteristics/index	Cheese					
	Rennet-curd			Acid-curd		
	Cheddar	Emmentaler	Camembert	Tvorog	Harzer	Fried
TN (%)	3.83^b^ ± 0.13	4.07^b^ ± 0.0.3	2.85^c^ ± 0.07	3.09^c^ ± 0.26	4.71^a^ ± 0.22	3.04^b^ ± 0.03
WSN (%)	1.01^b^ ± 0.13	0.60^bd^ ± 0.11	1.39^b^ ± 0.27	0.47^bd^ ± 0.34	3.31^a^ ± 0.19	1.62^bc^ ± 0.52
AN (%)	0.13 ± 0.02	0.08 ± 0.01	0.17 ± 0.04	0.14 ± 0.09	0.26^a^ ± 0.09	0.05^b^ ± 0.01
WSN/TN (%)	26.29^bc^ ± 2.53	14.80^bd^ ± 2.64	49.27^ac^ ± 10.92	13.82^bd^ ± 9.43	70.45^a^ ± 4.35	54.27^ac^ ± 16.64
AN/TN (%)	3.37 ± 0.45	2.04 ± 0.35	6.04 ± 1.67	4.35 ± 2.41	5.57 ± 2.06	1.64 ± 0.05

*TN* total nitrogen, *WSN* water soluble nitrogen, *AN* ammonium nitrogen

^a–d^Statistically significant differences between means (*p* ≤ 0.05) marked with different letters in rows

The content of ammonium nitrogen (AN) was the highest in harzer cheese and the lowest in fried cheese. In acidified short ripened fried cheese, the application of frying process resulted in the inhibition of the proteolysis, and thus, the value of AN reached the lowest value. The ammonium nitrogen indicates the deep proteolytic changes, leading to amino acid deamination and origin of ammonium (Chen et al. 2012). The depth of ripening described by AN/TN ratio was similar for all the assessed cheeses. Chen et al. (2012) reported that the amount of ammonium nitrogen in camembert was from 0.12 to 2.14 mg g^−1^ of dry matter.

The content of biogenic amines in the assessed cheeses was differentiated (Table 3), but the differences were not statistically significant, with the exception of their significant higher level in acid-curd ripened cheeses (harzer).

**Table 3:** Biogenic amine content in cheeses ($\bar{x}$ ± sd; mean values ± standard deviation)

Amines (mg kg^−1^)	Cheese					
	Rennet-curd			Acid-curd		
	Cheddar	Emmentaler	Camembert	Tvorog	Harzer	Fried
Cadaverine	1.60^b^ ± 0.28	4.33^b^ ± 1.79	2.43^b^ ± 0.80	7.35^b^ ± 3.64	377.5^a^ ± 155.0	4.20^b^ ± 2.42
Tyramine	5.78^b^ ± 1.78	67.58^b^ ± 56.31	6.23^b^ ± 2.88	7.49^b^ ± 3.16	275.5^a^ ± 107.9	6.90^b^ ± 1.75
Putrescine	2.67^b^ ± 0.78	67.02^b^ ± 6.73	6.47^b^ ± 3.13	6.17^b^ ± 3.49	281.33^a^ ± 114.9	3.11^b^ ± 1.03
Tryptamine	0.90^b^ ± 0.15	1.21^b^ ± 0.60	0.92^b^ ± 0.54	1.56^b^ ± 0.64	48.91^a^ ± 19.99	1.33^b^ ± 0.27
Histamine	5.80^b^ ± 3.64	2.45^b^ ± 1.12	1.08^b^ ± 0.25	3.01^b^ ± 2.01	24.08^a^ ± 1.71	1.08^b^ ± 0.76
Spermidine	2.29^b^ ± 0.99	1.67^b^ ± 0.48	2.82^b^ ± 1.54	1.86^b^ ± 0.43	7.74^a^ ± 1.09	5.41^ab^ ± 2.01
2-Phenylethylamine	2.08 ± 1.13	8.76 ± 6.85	0.87 ± 0.52	0.92 ± 0.92	5.30 ± 0.32	1.40 ± 0.77
Spermine	2.79^b^ ± 1.55	1.89^b^ ± 0.14	1.02^b^ ± 0.04	1.21^b^ ± 0.32	5.70^a^ ± 1.48	2.17^b^ ± 0.69
Total amines	21.12^b^ ± 6.34	153.01^b^ ± 124.20	20.82^b^ ± 5.37	28.36^b^ ± 12.77	1020.4^a^ ± 395.6	23.43^b^ ± 1.10

^a,b^Statistically significant differences between means (*p* ≤ 0.05) marked with different letters in rows; *n* = 18

The highest level of cadaverine was observed for harzer cheese (377.5 mg kg^−1^), and in all the other analysed cheeses, the amine level was lower than 10 mg kg^−1^, with the lowest value for cheddar. According to Fiechter et al. (2013), this amine is predominant in harzer cheese and can occur in quantity of 1268 mg kg^−1^. Standarová et al. (2010) described that Olomouc tvorogs also contained, among other amines, the highest level of cadaverine, up to 2413 mg kg^−1^. According to European Food Safety Authority (2011), fresh cheeses can contain from 10.7 to 45 mg kg^−1^ of cadaverine and the hard cheeses from 47.8 to 83.5 mg kg^−1^; but in some kinds of hard cheeses, the amine level can reach as much as 3170 mg kg^−1^. Research of Sawilska-Rautenstrauch et al. (2010) showed that cadaverine content in camembert is lower than 2.6 mg kg^−1^. In emmental cheeses, this amine occurs in the range 0.22–44 mg kg^−1^ (Pillonel et al. 2003).

The largest amount of tyramine was also found in harzer cheese, whereas emmental contained four times less, and the other analysed cheeses contained approximately 7 mg kg^−1^ of this amine (Table 3). According to Pillonel et al. (2003), emmental cheeses contain 8.6–403 mg kg^−1^ of tyramine. Much higher level of this amine was stated by Standarová et al. (2010) in Olomouc cheeses (1058 mg kg^−1^) and by Fiechter et al. (2013) in harzer cheese (516 mg kg^−1^). European Food Safety Authority (2011) reported that this amine content can reach, in fresh cheeses 48 mg kg^−1^, in hard cheeses 65.5 mg kg^−1^, and in acid-curd cheeses 55.3 mg kg^−1^; although much higher amounts were observed in some cheeses—in cheddar 910 mg kg^−1^ and 140 mg kg^−1^ in camembert.

Putrescine was located in the third place, when the amounts of biogenic amines were taken into account, for analysed cheeses. The highest amount of putrescine was observed in harzer cheese, and then in emmental and the last was cheddar (Table 3). The subsequent amounts of putrescine in cheeses are presented in papers: Olomouc tvorogs—767 mg kg^−1^, harzer cheese—802 mg kg^−1^, fresh cheeses from 5.5 to 41.3 mg kg^−1^, hard cheeses from 26.6 to 65.5 mg kg^−1^, acid-curd cheeses—335 mg kg^−1^, camembert 3.3 mg kg^−1^, and gouda up to 112.9 mg kg^−1^ (Sawilska-Rautenstrauch et al. 2010; Standarová et al. 2010; European Food Safety Authority 2011; Fiechter et al. 2013).

The contents of the remaining assessed biogenic amines (tryptamine, histamine, spermidine, 2-phenylethylamine, and spermine) ranged from 0.87 mg kg^−1^ (2-phenylethylamine in camembert cheese) to 48.91 mg kg^−1^ (tryptamine in harzer cheese) (Table 3).

The results obtained by other researchers revealed that camembert cheese can contain at about 30 mg kg^−1^ of histamine, Olomouc cheeses 411 mg kg^−1^ at average, different hard type cheeses such as emmental and cheddar from 65 to 672 mg kg^−1^, blue-veined cheeses even as much as 1850 mg kg^−1^, and acid-curd cheeses at approximately 55 mg kg^−1^ (Pillonel et al. 2003; Standarová et al. 2010; European Food Safety Authority 2011). Histamine is present in cheeses in the smaller amount than cadaverine, tyramine, and putrescine (European Food Safety Authority 2011). The contents of spermidine, spermine, and 2-phenylethylamine in different cheeses types are on average: approximately 16 mg kg^−1^; 48 mg kg^−1^, and 11 mg kg^−1^, respectively (Standarová et al. 2010; European Food Safety Authority 2011).

As the assessment results revealed (Table 3), the harzer cheese contained over 1000 mg kg^−1^ of biogenic amines, the emmental almost 7 times less, whereas other cheeses (cheddar, camembert, tvorog, and fried) contained below 30 mg kg^−1^.

Biogenic amines can pose a serious threat to human health, or even life when consumed in high amounts during one serving (European Food Safety Authority 2011). Cheeses are the second, after fish, main source of these compounds in human diet. In some countries, the limits for histamine consumption have been introduced, i.e., 200 mg kg^−1^ of fish, similarly for meat and meat products 100–200 mg kg^−1^, whereas limits for cheese consumption are non-existent (European Food Safety Authority 2011). This assessment measured cheeses were not a treat for the human because of histamine content as its levels were lower than 25 mg kg^−1^ of cheese.

The considerable differences in biogenic amines levels were obtained even for the same kind of cheese. The amounts for pecorino were observed from 4.4 to 2558 mg kg^−1^, for smear cheeses from 1000 to 1184 mg kg^−1^, for hard cheeses from 940 to 1030 mg kg^−1^, for blue-veined cheeses from 21 to 1516 mg kg^−1^, and for acid-curd cheeses as much as 2140 mg kg^−1^ (Bonczar et al. 2017).

Since it has been found that some biogenic amines can show a synergy effect, EFSA established that they pose no threat to human health when one serving dose of 50 mg of histamine and 600 mg of tyramine is consumed. Some literature data showed that cheeses are the main source of cadaverine in the diet (European Food Safety Authority 2011), so this amine should also be taken into account during establishing the threshold limits of biogenic amines for cheeses.

Among the assessed acid-curd cheeses (tvorog and harzer), the amounts of cadaverine, tyramine, and putrescine were in predominance, while in rennet-curd cheeses (emmental and camembert)—tyramine and putrescine; whereas in cheddar—histamine and tyramine.

The results of many research assessments showed that microorganisms present in cheeses were the main factors influencing on proteolysis range and on biogenic amines content (Curtin and McSweeney 2004; Upadhyay et al. 2004; European Food Safety Authority 2011, Fuentes et al. 2015, Hong-Xin et al. 2015).

The obtained results confirmed that opinion what can be seen when analysing the correlation coefficients (Table 4). The total aerobial bacteria count was correlated positively and statistically meaningful to the contents of water soluble nitrogen (WSN), to the ammonium nitrogen (AN), spermine, histamine, and spermidine contents; and the coefficients ranged from *r* = 0.48 to *r* = 0.75. The total aerobial bacteria count was not correlated statistically to other assessed individual bioamine contents.

**Table 4:** Correlation coefficients between assessed parameters

Parameter	2	3	4	5	6	7	8	9	10	11	12	13	14	15
1. Water	**− 0.93**	**− **0.24	**− **0.12	0.08	0.22	0.08	0.29	0.20	0.20	0.30	0.21	0.26	**− **0.20	0.03
2. Fat		**− **0.16	**− **0.03	**− **0.28	**− **0.41	**− **0.22	**− 0.51**	**− **0.44	**− **0.43	**− 0.52**	**− 0.52**	**− 0.53**	0.03	**− **0.35
3. Protein			0.33	**0.28**	0.49	0.32	**0.61**	**0.66**	**0.64**	**0.59**	**0.68**	0.37	0.40	**0.61**
4. pH				0.41	**0.65**	**0.28**	**0.52**	**0.54**	**0.53**	**0.52**	**0.51**	0.39	0.28	0.42
5. Bacteria count					**0.55**	**0.75**	0.01	0.01	0.00	0.03	**0.50**	**0.48**	0.14	**0.71**
6. WSN						**0.56**	**0.65**	**0.59**	**0.58**	**0.64**	**0.73**	**0.81**	0.11	**0.72**
7. AN							0.17	0.15	0.16	0.19	**0.55**	0.40	0.11	**0.57**
8. Cadaverine								**0.94**	**0.94**	**0.99**	**0.78**	0.43	0.14	0.37
9. Tyramine									**1.00**	**0.94**	**0.72**	0.34	0.45	0.34
10. Putrescine										**0.94**	**0.72**	0.33	0.45	0.33
11. Tryptamine											**0.79**	0.44	0.15	0.39
12. Histamine												**0.54**	0.17	**0.66**
13. Spermidine													**− 0.58**	**0.76**
14. 2-Phenylethylamine														0.19
15. Spermine														

Correlation coefficients in bold were statistically significant *p* ≤ 0.05

Also pH is mentioned in references as meaningful factor influencing proteolysis in cheeses and stimulating origin of some amines (European Food Safety Authority 2011; Chen et al. 2012). As showed the data presented in Table 4, pH of cheeses was statistically positively correlated (*p* ≤ 0.05) to contents of WSN, AN, and individually to histamine, tyramine, putrescine, cadaverine, and tryptamine contents. Chen et al. (2012) also showed the high positively correlated coefficient between pH and water soluble and ammonium nitrogen contents in cheeses.

Total protein content in cheeses was statistically positively (*p* ≤ 0.05) correlated to contents of histamine, tyramine, putrescine, cadaverine, spermine, and tryptamine, whereas water soluble nitrogen content to spermidine, spermine, histamine, cadaverine, tryptamine, tyramine, and putrescine contents.

Total fat content in assessed cheeses was statistically negatively (*p* ≤ 0.05) correlated to contents of cadaverine, tryptamine, histamine, and spermidine, what suggested that fat is not favouring the biogenic amine formation. Indeed, water activity in cheese decreases when content of fat increases. Parallel to the growth of fat content in cheeses, the water activity of the product is falling which inhibits proteolytic bacteria and reduces accessibility of free amino acids for bioamine formation (Benkerroum 2016). Although, from the other hand, the oxidation of fat can lead to aldehydes and ketones formation (Li et al. 2013) which, through amination and/or transamination, can form biogenic amines (Benkerroum 2016).

## Conclusions

It was found that the acid-curd short ripened cheese (harzer) was characterized by the greatest range of proteolysis and the biogenic amines content in comparison to other rennet- and acid-curd cheeses. The range of proteolysis in fresh acid-curd cheese (tvorog) and rennet-curd long ripened emmental were comparable which confirmed the influence of many different factors, besides the ripening and/or ripening time, on proteolysis. In the assessed acid- and rennet-curd cheeses, the dominant amines were subsequently: cadaverine, tyramine, and putrescine with except of cheddar where the histamine was predominant. The high and statistically positively combined correlation coefficients showed the dependence of proteolysis range and the biogenic amines content on pH and total bacteria count in cheeses. The negative and statistically meaningful correlation coefficients between fat and some biogenic amines contents showed the inhibitive influence of fat on some amines formation in cheeses.
